# Impact of lifestyle on health-related quality of life among young university students: a cross-sectional study

**DOI:** 10.1590/1516-3180.2021.0138.R2.120321

**Published:** 2021-07-16

**Authors:** Guillermo García-Pérez-de-Sevilla, Enrique Alonso Pérez-Chao, Helios Pareja-Galeano, Eva María Martínez-Jiménez, Marta de-la-Plaza-San-Frutos, Beatriz Sánchez-Pinto-Pinto, Carlos Romero-Morales

**Affiliations:** I PT. Physiotherapist and Professor, Department of Physiotherapy, Faculty of Sport Sciences, Universidad Europea de Madrid, Villaviciosa de Odón, Madrid, Spain.; II PT. Physiotherapist and Professor, Department of Physiotherapy, Faculty of Sport Sciences, University Alfonso X, Villanueva de la Cañada, Spain.; III PT. Physiotherapist and Professor, Department of Physical Education, Sport and Human Movement, Autonomous University of Madrid, Madrid, Spain; IV PT, PhD. Professor, Department of Physiotherapy, Facultad de Enfermería, Fisioterapia y Podología, Universidad Complutense de Madrid, Madrid, Spain; V PT, PhD. Physiotherapist and Professor, Department of Physiotherapy, Faculty of Sport Sciences, Universidad Europea de Madrid, Villaviciosa de Odón, Madrid, Spain; VI MD. Physician, Physical Medicine and Rehabilitation Unit, Hospital Universitario Puerta de Hierro Majadahonda, Majadahonda, Spain.; VII PT, PhD. Physiotherapist, Department of Physiotherapy, Faculty of Sport Sciences, Universidad Europea de Madrid, Villaviciosa de Odón, Madrid, Spain

**Keywords:** Quality of life, Public health, Lifestyle, Students, Health related quality of life, Disease prevention, Community health, Physical activity

## Abstract

**BACKGROUND:**

Lifestyle is strongly involved in the pathogenesis and progression of non-communicable diseases, and has a great impact on quality of life. The goal of the present study was to analyze the lifestyle and body composition (BC) of young university students during the pandemic, and their relationship with health-related quality of life (HrQoL).

**DESIGN AND SETTING:**

Observational cross-sectional study conducted in the Universidad Europea de Madrid, Spain.

**METHODS:**

A total sample of 56 healthy university students was recruited. Activity, sitting time, adherence to Mediterranean diet and BC were measured.

**RESULTS:**

Regarding BC, only 5% and 10.7% of the subjects had health risk values for waist circumference and waist-to-height ratio, respectively. The mean daily sitting-time was 8.26 hours, while 19.64% of the subjects spent . 10 hours per day sitting. 92.86% of the subjects complied with the World Health Organization 2020 physical activity recommendations. The mean PREDIMED score was 7.41, while 51.8% of the subjects had low adherence to the Mediterranean diet. Regarding HrQoL, 22 subjects (39.2%) and 26 subjects (46.4%) were in the lowest quintile of physical component summary and mental component summary, respectively, according to the reference values for their age range. There was a negative correlation between physical function and sitting time (r = -0.38).

**CONCLUSIONS:**

There were high levels of sedentary behavior and low HrQoL values, with a negative moderate correlation between these variables. The findings from the present study especially highlight the importance of implementing public health programs targeting reduction of sitting time among university students.

## INTRODUCTION

Lifestyle, through epigenetic mechanisms, is strongly involved in the pathogenesis and progression of noncommunicable diseases (NCDs),^1^ and has a great impact on quality of life.^2^ In this order, physical inactivity and obesity are, respectively, the two lifestyle risk factors most associated with the development of NCDs.^3^ Physical inactivity is the fourth largest risk factor for mortality worldwide,^3^ and it is associated with the comorbidities of overweight and obesity. People with these two conditions account for 27% of type 2 diabetes mellitus (T2DM) cases worldwide and 30% of ischemic heart disease cases.^4^ Consequently, the World Health Organization (WHO) has instituted the target of reducing the prevalence of physical inactivity by 15% worldwide by 2030.^5^

Every extra hour of daily sedentary behavior has a negative impact on health.^6^ Compared with subjects who sit for 6 hours a day, those who sit for 8 hours have a 14% higher cardiovascular risk and those who sit for 10 hours a day, 29%.^7^ There is strong evidence that individuals who maintain sedentary behavior over time have greater all-cause mortality, as well as several negative health-related outcomes. This is more pronounced among physically inactive people, given that physical activity attenuates mortality risk. Higher physical activity levels among highly sedentary individuals are thus required.^8^

The possible pathways to negative health-related outcomes facilitated by a sedentary lifestyle include promotion of increased oxidative stress. This is a strong precursor of endothelial dysfunction and gives rise to greater release of free fatty acids in the bloodstream, thus favoring development of cardiovascular diseases (CVDs).^9^ In addition, a sedentary lifestyle causes insulin resistance^10^ and promotes accumulation of visceral fat.^11,12^

Previous studies have reported that each additional hour of sedentary time is associated with greater gain in body mass index (BMI) and waist circumference (WC).^8^ A sedentary lifestyle can lead to development of obesity, which is the second highest risk factor for lifestyle-related premature death, after physical inactivity.^13^ Moreover, overweight and obesity cause insulin resistance^14,15,16^ and promoting deposition of ectopic lipids or visceral fat in different organs.^17^ This interferes with the normal function of organs, which thus increases the risk of many NCDs, especially CVD and T2DM.^18^ Physical activity is essential for achieving energy balance and weight control, thereby enabling maintenance of healthier body composition (BC) with an acceptable ratio of fat and muscle.^19^ It also protects against numerous NCDs,^20,21^ because it has a systemic dose-dependent anti-inflammatory effect.^6,22,23^

Regarding nutritional habits, adherence to the Mediterranean diet has been shown to have significant inverse associations with all-cause mortality^24^ and with the prevalence of CVD and T2DM, which are related to the anti-inflammatory properties of this diet.^25,26^ Severe acute respiratory syndrome coronavirus 2 (SARS-CoV-2) was discovered in Wuhan, Hubei province, China, in a pneumonia epidemic in January 2020.^27^ On January 30, 2020, it was declared by WHO to be a global health emergency^28^ and, since then, the virus has spread throughout the world, causing more than a million deaths.

The coronavirus disease 2019 (COVID-19) pandemic has affected and overwhelmed healthcare systems in unprecedented ways. Consequently, pending mass and effective vaccination, social distancing is the main preventive measure against SARS-CoV-2. Social distancing implies massive reorganization of society and lifestyles.^29^ During the period of confinement experienced between March and May 2020 in many countries, the number of people who practiced physical activity decreased by approximately 30%^30^ and the number of hours spent sitting increased by 28.6%.^31^ However, adherence to the Mediterranean diet was not significantly affected.^32^

NCDs generally develop in the elderly population, but lifestyle behaviors become established during young adulthood, when individuals do not perceive that an unhealthy lifestyle implies a higher health risk.

There is great variability in the dietary patterns of university students, in part because there are many different questionnaires assessing the adherence to the Mediterranean diet.^33^ Generally, university students have a low adherence to the Mediterranean diet.^34,35^ Regarding physical activity levels, university students normally are highly active, but also report high amounts of sedentary behavior.^36,37^ Students with higher levels of physical activity usually report greater levels of health-related quality of life (HrQoL), especially regarding the mental component summary (MCS).^38,39,40^

Further research on young adulthood is needed in order to ascertain the impact of lifestyle on HrQoL in young populations.

## OBJECTIVE

We aimed to analyze the lifestyle and BC of young university students during the pandemic, and their relationship with HrQoL. We hypothesized that physical activity levels, sedentary behavior, unhealthy BC and poor adherence to the Mediterranean diet would have moderate negative associations with HrQoL.

## METHODS

### Design

An observational cross-sectional study was developed, following the STROBE (strengthening the reporting of observational studies in epidemiology) guidelines, between October and November 2020 at Universidad Europea de Madrid, in Spain.

### Settings and participants

A total sample of 56 healthy young students was recruited at Universidad Europea de Madrid: age 23.5 ± 3.4 years; height 175 ± 8.1 cm; and body mass 68.3 ± 10.2 kg. The participants were recruited via e-mail between October and November 2020. The potential participants were reassured that nonparticipation would not have any consequences. A code was assigned to participants, prior to statistical analysis, thus guaranteeing the confidentiality of their data.

The inclusion criteria were that the participant needed to be a student at the Universidad Europea de Madrid^34^ and be between 18 and 34 years of age.^41^ The exclusion criteria were situations of: 1) having a chronic disease;^42^ 2) undergoing pharmacological therapy;^42^ or 3) having any condition that led to development of pain or any disturbances during physical exercise.^42^

### Ethical considerations

The current study was approved by the Research Ethics Committee of Universidad Europea de Madrid, under the protocol number CIPI/20/16, on September 30, 2020, and also respected the Helsinki guidelines at all times. All the participants read and signed an informed consent statement before becoming part of this investigation.

### Measurements

- *Physical activity level and sitting time* were measured using the Global Physical Activity Questionnaire (WHO, Geneva, Switzerland) (GPAQ), a validated tool that was developed by WHO for estimating physical activity levels (MET-minutes/week) in diverse countries around the world. This questionnaire makes it possible to see whether the subjects are complying with the 2020 WHO physical exercise recommendations, and whether they are spending large amounts of time seated. The amount of sitting time associated with greater all-cause mortality among adults varies from 6 to 8 hours per day, so we established > 420 minutes/day as the health risk threshold for this study.^4,8,43,^44^,45^
- *Adherence to the Mediterranean diet* was measured using the PREDIMED questionnaire (Schroder et al., Barcelona, Spain).^43^ High adherence is considered to be shown by scores > 10 points; medium adherence, 8–9 points; and low adherence < 7 points. Having high adherence to the Mediterranean diet brings strong protection against CVD.^44^
- *HrQoL* was measured using the short-form-36 (SF-36) health survey, version 2 (Alonso et al., Barcelona, Spain),^45^ which gives scores from 0 (worst health status) to 100 (best health status) in eight sections: physical function (PF), role physical (RP), bodily pain (BP), general health (GH), vitality (VT), social functioning(SF), role emotional (RE) and mental health (MH). The eight sections are regrouped into two main components: physical component summary (PCS) and mental component summary.^41^ The mean values for PCS and MCS are: 55.02 and 51.47 for men aged 18–24 years; 54.96 and 51.53 for men aged 25–34 years; 54.58 and 48.92 for women aged 18–24 years; and 53.87 and 49.62 for women aged 25–34 years.^41^
- *Body composition:* height, weight and WC were measured using a stadiometer (in cm; Ano Sayol SL height rod, Barcelona, Spain), a scale (in kg; Asimed T2 scale, Barcelona, Spain) and a tape (in cm), respectively. BMI and the waist-to-height ratio (WtHr) were then calculated. The values associated with an exponential risk of developing CVD and T2DM are WC > 102 cm in men and > 88 cm in women,^46^ and WtHr > 0.5 in men and women.^47^ WC and WtHr are highly correlated with visceral adipose tissue, which is the most clinically relevant adiposity variable, because of its association with the increased risk of NCDs. In contrast, BMI has a poor correlation with visceral adipose tissue.^47,48^ However, individuals with high BMI are at greater risk of developing high visceral adipose tissue levels, compared with individuals with healthy BMI.^49^

### Statistical analysis

Frequencies (sample size and proportion of samples) were assessed for categorical variables (adherence to Mediterranean diet and sitting time). The Kolmogorov-Smirnov test was used to assess the normality of distribution.^50^ A descriptive analysis was developed for all the subjects using the mean ± standard deviation (SD) to describe the parametric data and the median ± interquartile range (IQR) for nonparametric data. The coefficient of variation (CV%) was calculated for all continuous variables. In addition, an independent-sample t test (in situations of normal distribution) or the Mann-Whitney U test (in situations of non-normal distribution) was applied to determine differences between high/low adherence to the Mediterranean diet and the remainder of the continuous variables (HrQoL, physical activity and BC).

The Spearman correlation test with 95% coefficient intervals was carried out to analyze the relationships between continuous variables. The magnitudes of correlations between continuous variables were qualitatively interpreted using the following criteria: trivial (r < 0.1), small (r = 0.1-0.3), moderate (r = 0.3-0.5), large (r = 0.5-0.7), very large (r = 0.7-0.9) and almost perfect (r > 0.9).^51^ Otherwise, correlation was interpreted as the observed magnitude. The statistical significance was set at an alpha level of < 0.05. All analyses were conducted using IBM SPSS for Windows, version 23 (IBM Corporation, Armonk, New York, United States).

## RESULTS

### Sociodemographic data of the sample

A total of 56 subjects aged 23.5 ± 3.4 years were analyzed. Their mean BMI was 22.15 ± 2.28 kg/m^2^. Regarding WC and WtHr, only three (5%) and six subjects (10.7%), respectively, had health-risk values.

The adherence to Mediterranean diet showed equality of distribution. Twenty-seven subjects (48.2%) showed medium-high adherence while the remaining 29 subjects (51.8%) showed low adherence. Analysis on sitting time revealed that 25 subjects were at risk (44.6%) while the remainder of the subjects were at low risk (n = 31; 55.4%). Regarding HrQoL, 22 subjects (39.2%) and 26 subjects (46.4%) were in the lowest quintiles of PCS and MCS, respectively, according to the reference values for their age range.

The descriptive analysis, Kolmogorov-Smirnov normality test and CV% for all the continuous variables is presented in **Table 1**.

**Table 1.** Descriptive analysis on all continuous variables

	Variable (n = 56)	Value	CV%	P-value of Kolmogorov-Smirnov test
	Waist circumference	76.00 ±9.50 (65.0-102.0)†	10	0.003
Body composition	Body mass index	22.15 ±2.28 (18.6-26.9)*	10	0.200
	Waist-to-height ratio	0.43 ±0.04 (0.35-0.59)†	9	0.001
Physical activity	Physical activity level	1880.37 ±738.90 (240.0-3440.0)*	39	0.200
	Sedentary time	420.00 ±127.50 (360.0-920.0)†	26	0.000
	Physical functioning	100.00 ±5.00 (50.0-100.0)†	8	0.000
	Role physical	100.00 ±43.80 (0.0-100.0)†	44	0.000
	Bodily pain	84.00 ±22.00 (20.0-100.0)†	25	0.000
	General health	72.58 ±16.56 (30.0-97.0)*	23	0.028
Health-related quality of life	Vitality	66.07 ±17.12 (20.0-100.0)*	26	0.070
	Social functioning	87.50 ±25.00 (37.5-100.0)†	17	0.000
	Role emotional	83.50 ±91.80 (0.0-100.0)†	71	0.000
	Mental health	80.00 ±20.00 (20.0-100.0)†	19	0.003
	Physical component summary	53.16 ±7.15 (35.4-67.4)*	13	0.200
	Mental component summary	48.75 ±18.60 (16.8-62.5)†	25	0.004

Values shown are *mean ± standard deviation (SD) (minimum-maximum) or †median ± interquartile range (IQR) (minimum-maximum). CV% = coef.ficient of variation.

### Correlations between body composition, lifestyle and HrQoL

The Spearman correlation test with 95% coefficient intervals was used to analyze possible correlations between continuous variables (Table 2). Three HrQoL variables, namely VT (P = 0.013), RE (P = 0.018) and MCS (P = 0.015), showed moderate correlations (range for r = 0.31-0.33) with WC. In turn, SF was moderately correlated (r = 0.27) with sitting time and there was a negative correlation between PF and sitting time (r = -0.38; moderate magnitude). In addition, trivial-small correlations (range for r = -0.15 to 0.23) were found between HrQoL variables and the remainder of the physical activity and BC variables.

**Table 2.** Relationship (Spearman correlation test) between continuous variables with 95% coefficient intervals

			Body composition			Physical activity	
	Variables		Waist circumference	Body mass index	Waist-to-height ratio	Physical activity level	Sitting time
Body composition	Waist circumference	r	-	0.62**	0.82**	0.19	-0.07
		P [95% CI]	-	0.000 [0.427, 0.759]	0.001 [0.723, 0.896]	0.146 [-0.070, 0.437]	0.968 [-0.328, 0.196]
	Body mass index	r	0.62**	-	0.56**	0.22	-0.12
		P [95% CI]	0.000 [0.427, 0.759]	-	0.000 [0.429, 0.761]	0.093 [-0.039, 0.462]	0.368 [-0.373, 0.145]
	Waist-to-height ratio	r	0.82**	0.56**	-	0.10	-0.10
		P [95% CI]	0.000 [0.723, 0.896]	0.000 [0.429, 0.761]	-	0.441 [-0.162, 0.358]	0.449 [-0.356, 0.164]
Physical activity	Physical activity level	r	0.19	0.22	0.10	-	-0.23
		P [95% CI]	0.146 [-0.070, 0.437]	0.093 [-0.039, 0.462]	0.441 [-0.162, 0.358]	-	0.078 [-0.471, 0.027]
	Sitting time	r	-0.07	-0.12	-0.10	-0.23	-
		P [95% CI]	0.968 [-0.328, 0.196]	0.368 [-0.373, 0.145]	0.449 [-0.356, 0.164]	0.078 [-0.471, 0.027]	-
Health-related quality of life	Physical functioning	r	0.16	0.18	0.18	0.06	-0.38*
		P [95% CI]	0.217 [-0.100, 0.412]	0.165 [-0.079, 0.430]	0.164 [-0.078, 0.430]	0.651 [-0.205, 0.319]	0.003 [-0.588, 0.136]
	Role physical	r	-0.07	-0.11	-0.10	-0.05	0.12
		P [95% CI]	0.594 [-0.229, 0.194]	0.398 [-0.367, 0.152]	0.453 [-0.356, 0.165]	0.684 [-0.314, 0.210]	0.353 [-0.141, 0.377]
	Bodily pain	r	0.06	-0.01	0.04	-0.15	0.18
		P [95% CI]	0.623 [-0.199, 0.324]	0.891 [-0.280, 0.245]	0.725 [-0.218, 0.307]	0.266 [-0.398, 0.116]	0.185 [-0.087, 0.423]
	General health	r	-0.05	-0.07	0.04	0.21	-0.07
		P [95% CI]	0.715 [-0.309, 0.216]	0.600 [-0.328, 0.195]	0.752 [-0.222, 0.303]	0.116 [-0.054, 0.450]	0.573 [-0.333, 0.190]
	Vitality	r	0.33*	0.16	0.22	0.16	0.04
		P [95% CI]	0.013 [0.073, 0.545]	0.224 [-0.102, 0.410]	0.101 [-0.044, 0.458]	0.218 [-0.100, 0.412]	0.755 [-0.302, 0.223]
	Social functioning	r	0.22	0.27*	0.17	0.15	0.04
		P [95% CI]	0.096 [-0.041, 0.460]	0.038 [-0.016, 0.504]	0.205 [-0.095, 0.416	0.245 [-0.110, 0.404]	0.733 [-0.219, 0.306]
	Role emotional	r	0.31*	0.11	0.21	-0.03	0.14
		P [95% CI]	0.018 [0.073, 0.545]	0.752 [-0.156, 0.364]	0.105 [-0.046, 0.456]	0.781 [-0.298, 0.227]	0.273 [-0.119, 0.396]
	Mental health	r	0.12	0.04	0.13	-0.09	0.06
		P [95% CI]	0.375 [-0.147, 0.372]	0.752 [-0.222, 0.303]	0.314 [-0.130, 0.386]	0.500 [-0.346, 0.175]	0.622 [-0.199, 0.324]
	Physical component summary	r	-0.12	-0.09	-0.07	0.06	-0.00
		P [95% CI]	0.368 [-0.374, 0.145]	0.469 [-0.352, 0.169]	0.601 [-0.328, 0.195]	0.625 [-0.200, 0.324]	0.979 [-0.266, 0.259]
	Mental component summary	r	0.32*	0.15	0.21	0.04	0.10
		P [95% CI]	0.015 [0.068, 0.541]	0.257 [-0.113, 0.401]	0.110 [-0.050, 0.453]	0.747 [-0.221, 0.303]	0.443 [-0.163, 0.358]

Between physical activity variables and the remainder of the continuous variables (BC and HrQoL), trivial-small correlations (range for r = -0.17 to 0.24) were found. On the other hand, large-very large correlations (range for r = 0.56-0.82) were found between BC variables (**Table 2**).

Nonsignificant differences in BC, physical activity and HrQoL were found between medium-high and low adherence to the Mediterranean diet (**Table 3**). The comparisons of BC, physical activity and HrQoL between risk and non-risk sitting times are displayed in **Table 4**.

**Table 3.** Differences in body composition, physical activity and health-related quality of life between medium-high and low adherence to the Mediterranean diet

	Variable	Medium-high adherence group (n = 27)	Low adherence group (n = 29)	P value
	Waist circumference (cm)	76.00 ±8.00 (67.0-92.0)†	76.00 ±12.50 (65.0-102.0)†	0.967‡
Body composition	Body mass index (%)	22.07 ±2.17 (18.6-26.8)*	22.22 ±2.41 (19.0-26.9)*	0.820
	Waist-to-height ratio	0.44 ±0.04 (0.3-0.5)†	0.43 ±0.05 (0.3-0.5)†	0.373‡
Physical activity	Physical activity level (MET-min/week) Sitting time (minutes/week)	2011.85 ±707.89 (800.0-3360.0)* 420.00 ±60.00 (360.0-920.0)†	1757.93 ±756.33 (240.0-3440.0)* 480.00 ±180.00 (420.0-900.0)†	0.201 0.195‡
	Physical functioning	100.00 ±0.00 (85.0-100.0)†	100.00 ±5.00 (50.0-100.0)†	0.106‡
	Role physical	100.00 ±25.00 (25.0-100.0)†	100.00 ±5.00 (50.0-100.0)†	0.312‡
	Bodily pain	84.00 ±22.00 (20.0-100.0)†	84.00 ±22.00 (32.0-100.0)†	0.839‡
	General health	73.88 ±17.42 (30.0-97.0)*	71.37 ±15.94 (42.0-97.0)*	0.576
Health-related	Vitality	66.48 ±21.29 (20.0-100.0)*	65.69 ±12.44 (45.0-85.0)*	0.865
quality of life	Social functioning	87.50 ±25.80 (37.5-100.0)†	87.50 ±18.80 (62.5-100.0)†	0.473‡
	Role emotional	100.00 ±100.00 (00.0-100.0)†	66.70 ±83.50 (00.0-100.0)†	0.677‡
	Mental health	80.00 ±24.00 (20.0-96.0)†	80.00 ±16.00 (56.0-100.0)†	0.615‡
	Physical component summary	54.09 ±5.69 (40.9-65.4)*	52.29 ±8.30 (35.4-67.4)*	0.352
	Mental component summary	44.60 ±20.80 (16.8-60.9)†	45.37 ±16.10 (24.3-62.5)†	0.883‡

Comparisons are between high (n = 27) and low (n = 29) adherence to the Mediterranean diet. *Mean ± standard deviation (SD) (minimum-maximum) or †Median ± interquartile range (IR) (minimum-maximum). Statistical significance was set at an alpha level of < 0.05 (*). ‡Mann-Whitney U test was used.

**Table 4.** Differences in body composition, physical activity and health-related quality of life between risk and non-risk sitting time

	Variable	Risk (n = 25)	Non-risk (n = 31)	P-value
	Waist circumference	75.00 ±10.00 (66.0-92.0)†	77.00 ±10.00 (65.0-102.0)†	0.967‡
Body composition	Body mass index	22.82 ±2.10 (19.0-26.9)*	22.41 ±2.41 (18.6-26.8)*	0.337
	Waist-to-height ratio	0.43 ±0.05 (0.3-0.5)†	0.44 ±0.05 (0.3-0.5)†	0.373‡
Physical activity	Physical activity level Sitting time	1689.60 ±807.75 (240.0-3440.0)* 560.00 ±135.00 (480.0-920.0)†	2034.19 ±649.22 (960.0-3360.0)* 420.00 ±0.00 (360.0-420.0)†	0.082 0.195‡
	Physical functioning	95.00 ±5.00 (85.0-100.0)†	100.00 ±0.00 (50.0-100.0)†	0.106‡
	Role physical	100.00 ±25.00 (00.0-100.0)†	100.00 ±75.00 (00.0-100.0)†	0.312‡
	Bodily pain	84.00 ±17.00 (51.0-100.0)†	74.00 ±23.00 (20.0-100.0)†	0.839‡
	General health	71.16 ±17.48 (37.0-97.0)*	73.74 ±15.98 (30.0-97.0)*	0.567
Health-related	Vitality	66.00 ±15.87 (40.0-100.0)*	66.12 ±18.33 (20.0-90.0)*	0.978
quality of life	Social functioning	87.50 ±25.00 (62.5-100.0)†	87.50 ±25.80 (37.5-100.0)†	0.473‡
	Role emotional	100.00 ±83.40 (00.0-100.0)†	66.70 ±100.00 (00.0-100.0)†	0.677‡
	Mental health	80.00 ±20.00 (60.0-96.0)†	80.00 ±24.00 (20.0-100.0)†	0.615‡
	Physical component summary	53.35 ±6.84 (37.6-67.4)*	53.00 ±7.51 (35.4-66.1)*	0.856
	Mental component summary	49.00 ±16.70 (25.4-58.4)†	43.40 ±19.80 (16.8-62.5)†	0.883‡

Comparisons are between risk (n = 25) and non-risk (n = 31) sitting time. *Mean ± standard deviation (SD) (minimum-maximum) or †median ± interquartile range (IQR) (minimum-maximum). Statistical significance was set at an alpha level of < 0.05 (*). ‡Mann-Whitney U test was used.

## DISCUSSION

The subjects analyzed in this study had healthy BC values, as only 5% and 10.7% of the subjects had health-risk values for WC and WtHr, respectively. Regarding lifestyle, 44.6% of the subjects spent more than seven hours a day sitting down, which is a predisposing factor for development of cardiometabolic diseases over the long term.^24,52^ Individuals who maintain sedentary behavior over time have the highest all-cause mortality.^8^ Levels of physical activity were high (1880.37 ± 738.90 MET-min/week), such that 92.86% of the subjects were complying with the WHO 2020 physical activity recommendations.^53^ This was similar to the results reported in other studies with a student population.^36,37^

Physical activity attenuates mortality risk, and higher physical activity levels are required among highly sedentary individuals.^8^ Each additional hour of sedentary time is associated with greater gain in BMI and WC.^8^ However, in this study, we found only trivial-small correlations for physical activity and sitting time in relation to BC. The mean daily sitting time was 8.26 hours, while 19.64% of the subjects spent > 10 hours per day sitting.^8^ This is of great importance because if these habits are maintained over time, the risk of developing NCDs increases,^3^ given that sedentary behavior is associated with loss of metabolic flexibility,^3^ higher oxidative stress, insulin resistance, inflammation and DNA damage.^54,55^ Self-reported estimates from other studies have indicated that university students spend an average of 7.29 hours per day sitting, which is similar to our results. In contrast, data from accelerometer-based studies have suggested that university students engage in 9.82 hours of sedentary behavior per day. Chastin et al. suggested that self-reports underestimate sedentary behavior, compared with accelerometer-based methods,^37^ so the subjects of our study may have been spending more time sitting that we thought.

Concerning nutritional habits, half of the subjects analyzed (51.8%) had low adherence to the Mediterranean diet. The mean PREDIMED score was 7.41, which denotes acceptable adherence to the Mediterranean diet, in line with data from the Spanish population that was previously reported.^26^ However, other studies on student populations reported lower adherence to the Mediterranean diet.^34,35^ Considering these data, it is necessary to seek strategies to reduce sitting time in the young population, in addition to promoting the Mediterranean diet, since the lifestyle habits developed in youth can affect health in old age. It was striking that 39.2% and 46.4% of the subjects were in the lowest quintiles of PCS and MCS, respectively, according to the reference values for their age range.^41^

We did not find any study reporting on HrQoL using the SF-36 questionnaire among students that we could compare with our results. However, several authors have argued that the negative impact on QoL among sedentary individuals and university students was related to foot health disorders.^56,57^ The low levels of HrQoL reported in the present study are probably related to the massive reorganization of our society and our lifestyle due to the COVID-19 pandemic.^29^

Regarding the relationship between BC and lifestyle variables and HrQoL, WC showed moderate correlations with VT, RE and MCS. This is not of great clinical relevance because most of the subjects had healthy values for WC. In turn, sitting time was negatively correlated with PF, which highlights the negative impact of sedentary behavior on health.^7^ However, taking > 7 hours of sitting time per day as a reference value for health risk, no significant correlations were found between sitting time and the other variables. Adherence to the Mediterranean diet did not show any significant correlation with HrQoL or BC, which is relevant because this young population may not see any motivation to engage in changes in this regard. Moreover, large correlations exist between WC and BMI, and very large correlations exist between WC and WtHr. It is known that WC is more associated with cardiometabolic diseases risk factors, as well as WtHr,^58^ compared with BMI. In fact, in other populations, WC is a better indicator of poor physical HrQoL than BMI.^59^

The aim of this study was to characterize the lifestyle of young students in a pandemic period. We mainly found that these subjects were complying with the 2020 WHO physical exercise recommendations^53^ and had healthy BC. However, half of them had a poor dietary pattern and spent too many hours sitting. According to several studies, during the current COVID-19 pandemic period, physical activity levels have decreased by 30%^30^ and daily sitting time has increased (28.6%),^31^ while adherence to the Mediterranean diet has not been affected.^32^ This indicates that there needs to be a focus on improving these two aspects of the lifestyle of the young population. We also wanted to analyze HrQoL and its correlations with lifestyle. Sitting time was the variable that most negatively affected HrQoL, and it was striking that 39.2% and 46.4% of the subjects were in the lowest quintiles of PCS and MCS, respectively, according to the reference values for their age range. These results might be related to the difficult times of uncertainty and restriction of mobility that we have been going through, in the context of the COVID-19 pandemic.

### Study limitations and future lines

Our sample was obtained from just one particular university in one particular city. It would be very useful to obtain information from more universities in different locations. In addition, studying the implementation of a strengthening program or physical activity in relation to the Mediterranean diet, both among healthy individuals and among individuals presenting any pathological condition (e.g. COVID-19, musculoskeletal disorders or metabolic diseases) would be very interesting. Lastly, no comparative data from before the pandemic were obtained and, thus, the PCS and MCS cannot be attributed to the COVID-19 pandemic.

## CONCLUSION

In this young student population, the results showed that the subjects generally had healthy BC, high physical activity levels, acceptable adherence to the Mediterranean diet, high levels of sedentary behavior and very low levels of HrQoL. Regarding the relationship between lifestyle and HrQoL, only trivial-small correlations were found. Regarding lifestyle, the findings of the present study especially highlight the importance of implementation of public health programs targeting reductions in sitting time among university students.
